# Evolutionary genomics and divergence of *Cacopsylla* species with a special focus on the apple proliferation vectors *Cacopsylla melanoneura* and *Cacopsylla picta*

**DOI:** 10.1186/s12864-026-12622-0

**Published:** 2026-03-19

**Authors:** Lapo Ragionieri, Liliya Štarhová Serbina, Erika Corretto, James M. Howie, Fernando Cruz, Tyler S. Alioto, Nicola Zadra, Tobias Weil, Gianfranco Anfora, Christian Stauffer, Lino Ometto, Omar Rota-Stabelli, Hannes Schuler

**Affiliations:** 1 Competence Centre for Plant Health, Free University of Bozen- Bolzano, Bozen-Bolzano, Italy; 2 Center for Integrative Biodiversity Discovery, Leibniz Institute for Evolution and Biodiversity Science, Museum für Naturkunde, Berlin, Germany; 3 Museum of Nature Hamburg, Leibniz Institute for the Analysis of Biodiversity Change, Hamburg, Germany; 4 Department of Ecosystem Management, Climate and Biodiversity, BOKU University, Vienna, Austria; 5 Center for Cancer Research, Medical University of Vienna, Vienna, Austria; 6 Centro Nacional de Análisis Genómico (CNAG), Baldiri Reixac 4, Barcelona, 08028 Spain; 7 Universitat de Barcelona (UB), Barcelona, Spain; 8 University of Trento, Center Agriculture Food Environment (C3A), Trento, Italy; 9 Centro Ricerca ed Innovazione, Fondazione Edmund Mach, San Michele all’ Adige, Italy; 10 Department of Biology and Biotechnology, University of Pavia, Pavia, Italy; 11 Faculty of Agricultural, Environmental and Food Sciences, Free University of Bozen-Bolzano, Bozen, 39100 Italy

**Keywords:** Psylloidea, Genome assembly, Mitochondrial genes, Time-calibrated phylogeny, Transposable elements, Apple proliferations, Overwintering behaviour

## Abstract

**Background:**

The psyllid genus *Cacopsylla* includes several species that act as vectors for phytoplasma-associated diseases affecting plantations across Europe. Among them, *Cacopsylla melanoneura* and *Cacopsylla picta* are the primary vectors of ‘*Candidatus* Phytoplasma mali’, the phloem-restricted bacterium responsible for Apple Proliferation disease in Europe. To explore whether vector competence in these species reflects shared ancestry or independent evolution, we assembled mitochondrial and draft nuclear genomes of Italian populations of *C. melanoneura* and *C. picta* and reconstructed time-calibrated phylogenies using 13 mitochondrial protein-coding genes from 12 *Cacopsylla* species.

**Results:**

Phylogenetic analyses revealed two major *Cacopsylla* clades (Clade I and II) whose divergence times range from the Early Miocene (18.4 MYA; 95% HPD: 10.8–27.5) to the Middle Miocene (12.7 MYA; 95% HPD: 9.7–16.0). Both *C. melanoneura* and *C. picta* are within Clade I, which is predominantly composed of univoltine species that overwinter on conifers. Within this clade, *Cacopsylla melanoneura* is more closely related to the plum psyllid *Cacopsylla pruni* than to the apple-associated *Cacopsylla picta* and *Cacopsylla mali*, the latter belonging to Clade II. Draft nuclear genomes revealed significant differences in size (438 Mb in *C. melanoneura vs.* 631 Mb in *C. picta*), largely attributed to repetitive elements. Comparative analyses of repetitive elements across *Cacopsylla* species revealed a recent expansion of transposable elements, particularly LINE elements, which were slightly more abundant in Clade I and contributed to the larger genome size observed in *C. picta*.

**Conclusions:**

Collectively, our findings provide the first genomic resources for *C. melanoneura*, *C. picta*, and several other phytoplasma-vectoring *Cacopsylla* species. We established a robust mitogenomic phylogeny with divergence estimated for this genus showing the presence of two clades with the representatives predominantly associated with different overwintering strategies. Our results further indicate that vectorial capacity in *Cacopsylla* reflects an independent evolutionary trajectory rather than a shared ancestral origin. This evolutionary framework advances our understanding of the biology and origin of vector competence in this agriculturally important group.

**Supplementary Information:**

The online version contains supplementary material available at 10.1186/s12864-026-12622-0.

## Background

Psyllids (Hemiptera: Psylloidea) are a group of small phytophagous insects comprising ~ 4,000 described species belonging to seven families [1]. They are members of Sternorrhyncha, a suborder which includes several other well studied plant pathogen vectors such as aphids (Aphidoidea), whiteflies (Aleyrodoidea) and scale insects (Coccoidea) [1, 2]. Like many phloem feeding hemipterans, various psyllid species can vector phytoplasmas. Phytoplasmas are obligate, cell wall-less, prokaryotic plant pathogens (Mollicutes) inhabiting the phloem sieve elements of their host plant, and are responsible for a variety of economically important tree and crop diseases [3–5] including European Stone Fruit Yellows, Pear Decline, and Apple Proliferation [5, 6]. Phytoplasmas alter the plant’s cellular processes by interfering with its ability to transport nutrients and water. This results in weakened and malformed structures, as well as in significant yield reductions [4–6].

Apple Proliferation (AP) is an important disease affecting apple trees, associated with the phytoplasma *‘Candidatus* Phytoplasma mali’ (hereafter, ‘*Ca*. P. mali’; for a review see Janik et al. [6]). Distinctive symptoms include characteristic shoots with a “witches’ broom” appearance and fruits of reduced size and quality [7]. Infected trees cannot be cured, and they must be eradicated, leading to significant yield losses [6, 8, 9]. The transmission of phytoplasma occurs when the vector insects feed on the phloem of infected plants, acquiring phytoplasma which must then pass through the insect’s digestive system, cross the gut barrier, replicate, and invade the salivary glands. When the infected psyllid later feeds on the phloem of a healthy apple tree, the pathogen can be transmitted thus establishing a new infection [10]. The genus *Cacopsylla* (Psyllidae) includes all known vectors for fruit tree phytoplasmas belonging to the phytoplasma group 16SrX [11, 12]. The main vectors of AP are *C. melanoneura* and *C. picta*, while other hemipteran species like the leafhopper *Fieberiella florii* (Stål, 1864), are playing only a minor role [13]. *Cacopsylla picta* (Föster, 1848) acts as the main vector of the AP in most European countries [14–16] while *C. melanoneura* (Föster, 1848) is the main vector in the areas of Northwestern Italy where *C. picta* is absent [14, 17, 18]. Due to variation in vector capabilities of these two psyllids, various environmental and genetic factors are expected to influence the rate of acquisition and transmission of ‘*Ca.* P. mali’. These factors include the infectious status of the host tree, the genetics of the psyllid vector [16, 19], the insect’s endosymbionts [20] and the phytoplasma itself [19].

*Cacopsylla*, like most other psyllid species, are host specific, with closely related psyllids tending to associate with closely allied host plant lineages [21, 22]. In temperate regions, many psyllid species migrate from their deciduous host plants to conifers, where they overwinter as adults or eggs [23]. In contrast, psyllids in tropical and Southern temperate regions are often associated with evergreen hosts and tend to remain on the same plant throughout the year, completing several generations [23]. Intriguingly, some *Cacopsylla* species can develop on multiple related host plants (i.e. Rosaceae), forming genetically distinct groups with differing fitness levels. This is exemplified by *C. melanoneura*, which appears to have at least two genetically distinct populations, associated either with apple trees or hawthorn [24].

The timing of *Cacopsylla* diversification has not yet been inferred using time-calibrated phylogenies, leaving the genus’s evolutionary history, particularly its historical biogeography and host-plant associations, poorly understood. To date, a Neighbor-Joining analysis based on partial *cytochrome oxidase I* (COI) gene sequences provided weak support for most deep clades, including those containing the AP vectors *C. melanoneura* and *C. picta*, or other *Cacopsylla* species like *C. mali* and *C. pruni* [25]. A more recent phylogeny, based on whole mitochondrial genomes that included several *Cacopsylla* species, was not time-calibrated and omitted both *C. melanoneura* and *C. picta*, limiting its utility for resolving the evolutionary relationships among key vectors of *‘Ca.* P. mali’ [26]. Genomic information for these two species, and psyllids more broadly, remains limited, with existing studies focusing mainly on mitochondrial genomes [27]. These studies suggest the existence of two distinct clades, which distinguish species with differences in overwintering behaviour [28], although the diversification, including at the genetic and genomic level, is not yet established.

A comprehensive understanding of a species evolutionary history requires investigating the evolution of the nuclear genome, which is shaped by various molecular mechanisms such as different classes of mutations, gene duplications, recombination, and horizontal gene transfer. In many species one major component involved in genome evolution and adaptation is represented by transposable elements [29]. Transposable elements (TEs) are mobile DNA sequences that can significantly influence the genome structure and its functions. In insects, the proportion of the genome occupied by TEs varies widely, reaching up to 60% in some species such as the migratory locust [30]. Although TE activity can have potentially deleterious effects on gene regulation, increasing evidence suggests that TEs also drive genomic innovation and can confer selective advantages to the host [31]. Comparative studies have revealed both similarities and differences in TE family diversity and abundance among insect genomes. Notably, significant variation in TE activity has been observed even among closely related species [32]. For example, within the order Hemiptera, contrasting TE profiles have been reported. In *Homalodisca vitripennis* (Hemiptera, Cicadellidae), LINEs and DNA transposons show a distinct divergence distribution, while SINEs and LTR elements are poorly represented. This pattern contrasts sharply with that of the pea aphid *Acyrthosiphon pisum* (Hemiptera, Aphididae), where SINEs dominate the TE content, and LINEs and LTRs are virtually absent.

To overcome the limited genomic resources available for *Cacopsylla* species, we present newly assembled mitochondrial genomes and draft nuclear genome assemblies of *C. melanoneura* and *C. picta*. The mitochondrial genomes, together with publicly available data from other *Cacopsylla* species, were used to reconstruct a robust, time-calibrated phylogeny of the genus, including several vector species with different host plant preferences. In parallel, we provide the first nuclear genome assemblies of *C. melanoneura* and *C. picta* and use them to investigate the role of repetitive elements in psyllid genome evolution. Given the established involvement of repetitive elements in adaptive evolution and gene regulation, we also performed a comparative analysis across multiple *Cacopsylla* species, with a particular focus on the AP vector species *C. melanoneura* and *C. picta*, to identify lineage-specific traits potentially associated with the accumulation of recently active TEs.

## Methods

### Psyllid collection, DNA extraction, library preparation and sequencing

A total of 20 individuals of each species, *C. melanoneura* and *C. picta*, were collected in 2015 using the beating tray method in apple orchards in Northeastern Italy, specifically from Laimburg (South Tyrol) and Bosentino (Trentino). Samples were preserved at − 20 °C in absolute ethanol. Total DNA was extracted from each individual, and those with higher quality and yield were selected for subsequent library preparation and sequencing. For *C. melanoneura*, DNA was extracted using the NucleoSpin Tissue Kit (Macherey-Nagel), and three paired-end libraries were constructed from two individual specimens and one pool of six individuals, with different fragment sizes (PE290, PE310, and PE700; Table S1). For *C. picta*, an enhanced DNA extraction protocol was used, involving overnight lysis with DTT and extraction with the KingFisher Cell and Tissue DNA Kit (Thermo Fisher Scientific). Two paired-end libraries were prepared, each from a single individual and with different fragment sizes (PE510 and PE750; Table S1).

All five libraries were sequenced on the Illumina HiSeq 2000 platform with a 251 bp insert size (PRJNA1312285). This yielded 57.9, 59.4, and 75.3 Gb of raw data for *C. melanoneura*, corresponding to genome coverages of 131×, 135×, and 171.74×, respectively. For *C. picta*, sequencing produced 121.5 and 74.9 Gb of raw data, with corresponding coverages of 192× and 119×.

### Nuclear genome size estimation in *Cacopsylla* species

Prior to the *de novo* assembly, the genome sizes of *C. melanoneura* (Table S2) and *C. picta* (Table S2) were estimated using a k-mer analysis of the raw reads of library PE700 and PE510, respectively, using *Jellyfish* v2.2.6 [33], *GenomeScope* v1.0 (*k* = 17) [34] and *SGA preqc* (*k* = 31) [35]. Additionally, using this approach we also estimated the genome size of *C. burckhardti* (PRJNA927338) and *C. jukyungi* (PRJNA927338), for which raw data with sufficient coverage are publicly available.

### Assembly of the nuclear genomes of *C. melanoneura* and *C. picta*

Adapter sequences were removed and reads were trimmed in the *Trim Galore* wrapper (http://www.bioinformatics.babraham.ac.uk/projects/trim_galore/) using *Cutadapt* [36]. Overlapping reads from the shorter fragment libraries were merged using *FLASH* [37], after which reads were filtered in *GEM-mapper* [38] (with up to 2% mismatches) against a contamination database including phiX, Univec sequences, *E. coli*,* Cacopsylla coccinea* mitochondrion complete genome (NC_027087.1) and 17 contaminant species’ genomes (detected independently for each *Cacopsylla* species, using with *Kraken* v0.10.5 [39] as present in ≥ 0.03% of the reads, see Table S3).

The *C. melanoneura* genome was assembled *de novo* using overlapping read-pairs from the PE700 library (detected with *FLASH*) using *DISCOVAR de novo* (experimental release v.51885) with default options [40, 41], after discarding all read-pairs shorter than 200 bp. Circular lines were discarded, and the *DISCOVAR* contigs were scaffolded with *BESST* v2.2.5 [42] using processed reads from all three PE libraries (PE290, PE310 and PE700) with the option *“--separate_repeats”* and discarding all contigs classified as repeats. Mis-assemblies were removed as in [43, 44], using reads from all PE libraries, after introducing breaks at genomic intervals calculated to have negative consistency scores. This resulted in shorter but more reliable scaffolds, which were then broken again into contigs. Then contig assembly was “unchaffed” by filtering out those shorter than the read length (250 bp), thus only retaining sequences long enough to contain at least a partial exon (251–500 bp). Finally, the filtered contigs were re-scaffolded in *BESST* v2.2.5 yielding a final draft assembly (Table S4).

The *C. picta* genome was assembled with a modified version of the *C. melanoneura* pipeline. Overlapping read-pairs from library PE510 were assembled with *DISCOVAR de novo* [40, 41], discarding both read-pairs shorter than 200 bp and circular lines. The *DISCOVAR* contigs were collapsed with *Redundans* [45] to reduce heterozygous haplotypes and repeats, thus accounting for high fragmentation. *Redundans* contigs were scaffolded with *BESST* v2.2.5 [42] using both PE libraries with the “--*separate_repeats*” option, yielding the final assembly draft (Table S5).

Estimates of genomic completeness were obtained for each genome using two methods. *KAT* [46] compared k-mers in the pre-processed reads (libraires PE290, PE310, PE700 for *C. melanoneura*, PE510 for *C. picta*) with those in the final genomes to estimate sequence completeness. To assess genome assembly quality, we used *BUSCO* v5.8.3 [47], which estimates the completeness of genomic data based on the presence of universal single-copy orthologs (OrthoDB database odb12).

### Repetitive element annotation and comparisons

The expected sample heterozygosity and genomic repetitiveness were estimated in *GenomeScope* v1.0 [34] (Table S2). We then conducted a comparative analysis of abundances, sequence length and genome percentage for multiple repetitive element classes, including retroelements (LINEs, SINEs, LTRs), DNA transposons, and interspersed repeats (sRNAs, DNA satellites, simple repeats) using *RepeatMasker* [48], by mapping masked reads to a *RepeatMasker* Combined Database (Dfam_Consensus-20181026, RepBase-20181026) with *RMBlastn* v2.6.0+ (command: *RepeatMasker scaffold.fa -species arthropoda -pa 3*). In addition, *RepeatModeler2* [49] with the option “–LTRStruct” was used to identify dispersed transposable elements. Subsequently, *RepeatMasker* was used to identify and mask interspersed repeats in the nuclear draft assemblies of *C. melanoneura* and *C. picta*, using the *RepeatModeler2* library option. Additionally, we identified satellites and putative satellites in the raw reads and included them in *RepeatModeler2* families for use in a *RepeatExplorer2* analysis. Briefly, the pipeline was run to characterize the repeatome [50, 51], including the *TAREAN* analysis within the *Galaxy* portal (https://repeatexplorer-elixir.cerit-sc.cz). For this, we randomly selected a total of 2,000,000 paired reads. Default options were selected, except that we set computing time to “long”, applied a filtration of the most abundant repeats, and set the threshold of the analysis to 0.001%.

Besides *C. melanoneura* and *C. picta* presented here, no nuclear genome assemblies are available for other *Cacopsylla* species. For some other species, sequencing reads are publicly available (Tables S6). To analyse the genomic content and compare nuclear repeats across *Cacopsylla* species, we used *dnaPipeTE* (https://github.com/clemgoub/dnaPipeTE*)*, a tool optimized for repeatome analysis from low-coverage sequencing data [52] that uses raw reads as input. Before analysis, we removed reads matching to mitochondrial DNA, known symbionts and contaminants. Repeat annotation was performed using the Dfam-RepeatMasker.lib database for Arthropoda, which included 6,828 entries as of May 2025. Repeat landscapes were generated using the dnaPTlandscape.sh script from the dnaPT_utils package (https://github.com/clemgoub/dnaPT_utils), with the -S and -U options enabled to visualize only repeat superfamilies and to exclude unclassified elements.

### Assembly of the mitochondrial genomes of *C. melanoneura* and *C. picta*

Mitochondrial genomes of *C. melanoneura* (PX243765) and *C. picta* (PX243766) were assembled and circularised in the *MitoZ* pipeline [53] operated in a *Singularity* container [54] using Illumina raw read libraries PE290 and PE750, respectively. Mitogenomes were annotated in *MITOS* [55] and *MITOS2* [56], a consensus annotation manually compiled, and the genomes visualised with annotations, GC content and coverages in *MitoZ* [53].

### Mitochondrial phylogeny and divergence time estimation

To test the phylogenetic placement of *C. melanoneura* and *C. picta* within the *Cacopsylla* genus, we conducted phylogenetic analyses using Bayesian inference (BI) in *BEAST* v2.7.5 [57, 58] and maximum likelihood (ML) in *IQ-TREE* v1.6.12 [59]. The best-fit partitioning schemes and substitution models for subsequent phylogenetic analyses were predicted with *ModelFinder* [59] implemented in *IQ-TREE* release 2.1.4b [59]. We repeated the analyses using three datasets: amino acid sequences, the corresponding nucleotide (codon) sequences, and nucleotide sequences with the third codon position excluded. The mitochondrial sequences of 13 protein coding genes (PCGs) from 12 *Cacopsylla* species, eight psyllids from other families, two aphids and two white flies (Table S6) were aligned using the MAFFT-L-INS-I algorithm [60] and concatenated using *catsequence* 1.3 (https://zenodo.org/record/4409153#.YmJYT35Byot).

The optimal substitution model for each partition of the BI analyses was determined using the model-averaging approach implemented in OBAMA [57] with all substitution models enabled for the amino acid data, and the bModelTest packages [61] for the nucleotide data. The BI analyses of 100 million MCMC generations, with trees sampled every 1,000 generations, were run on the CIPRES platform [62] using BEAST [57]. Random starting trees were assigned to each partition. Divergence time estimation was conducted using the Most Recent Common Ancestor (MRCA) approach, incorporating various calibration points using *BEAUTi* [57] under an Optimized Relaxed Clock model. A time constraint derived from the posterior estimate of Li et al. [63] was applied to the split between Psylloidea and its sister clade (Aleyrodoidea + Aphidoidea) set at 269 MYA and modeled as a normal prior distribution (mean = 269, S = 5.9). Outgroup taxa from Aleyrodoidea and Aphidoidea were represented by *Aleurodicus dugesii* + *Bemisia tabaci* and *Aphis gossypii* + *Myzus persicae*, respectively. To calibrate the Psylloidea crown node, we used the Eocene fossil *Eogyropsylla paveloctogenarius* (Aphalaridae; 41.3–47.8 MYA), the oldest known representative of Psylloidea s.str [64]. The youngest age (41.3 MYA) was applied as a soft minimum using a log-normal prior (M = 3.67, S = 0.32, offset = 41.3), with *Lanthanaphalara mira*, an extant member of the same family, used to define the calibrated node. An additional calibration was applied to a tribal-level node within the family Liviidae (Paurocephalini), using the Early Miocene fossil *Melanastera casca* (20.4–13.8 MYA), a congener of the extant *M. paucipunctata* [65]. The youngest age (13.8 MYA) was treated as a soft minimum, implemented as a log-normal prior (M = 3.3, S = 0.39, offset = 13.8).

To select the tree prior (Yule *vs.* birth–death models), we performed a marginal likelihood estimation (MLE) using *Tracer* [66, 67]. The marginal likelihood of both priors was calculated with 1,000 path steps, running chains for 1 million generations, and sampling log likelihood every 1,000 cycles. This analysis was conducted using *BEAST* v1.10.4 [68]. We calculated the Bayes factor (BF) as twice the natural logarithm (2lnBF) with respect to the highest-likelihood model, considering BF values > 10 as significantly favouring one model over another. The remaining prior parameters for both Yule and birth-death models were kept unchanged. The results were evaluated in *Tracer* v1.7.1 [66], using the effective sampling size (ESS > 200) criterion, and the first 30% of samples were discarded as burn-in. The tree files were then combined using *LogCombiner* v2.4.5, and the parameter values were annotated to the Maximum Clade Credibility (MCC) tree using *TreeAnnotator* v2.4.5. Nodal support was assessed by posterior probabilities (PP).

ML and BI trees were visualized using *FigTree* v1.4.4 (https://github.com/rambaut/figtree). All figures were edited in *GIMP* v. 2.10.38.

## Results

### Nuclear genomes of *C. melanoneura* and *C. picta*

Based on raw sequencing reads, *C. melanoneura* has an estimated genome size of 438.67 Mb, while *C. picta* has a larger genome with 632.08 Mb (Table S2). The other species with sufficient and public available raw reads data showed intermediate genome sizes: 528.10 Mb for *C. jukyungi* and 549.12 Mb for *C. burckhardti*.

The final assembly of the *C. melanoneura* genome yielded a 688 Mb sequence, in a total of 678,097 contigs and 483,574 scaffolds with N50 values of 1.53 kb and 3.92 kb, respectively (Table S4). The assembled genome size was thus inflated relative to a priori estimates of 438.7–473.8 Mb (Table S2). The BUSCO completeness analysis revealed 32.9% of genes recovered as complete (of which 4.6% were duplicates), 31.4% as fragmented and 35.7% missed (Fig. S1). As expected by using data from a pool of individuals, this genome appears to be highly heterozygous (2.51%) and repetitive (estimated at 57.28%) a considerable number of *k*-mers were missed in the heterozygous and homozygous regions, and several artificial duplications and repeats emerged due to the non-perfect collapsing of haplotypes.

The assembled *C. picta* genome was larger, with 739.04 Mb and shows contig and scaffold N50s of 2.22 Kb and 6.3 Kb respectively (Table S5). The BUSCO completeness analysis indicated 30.1% of genes complete (of which 0.7% were duplicates), 33.1% fragmented (i.e. partial matches) and 36.8% missed. Because the genome was heterozygous (1.37%) and repetitive (estimated at 67.1%), there were some difficulties collapsing alleles in heterozygous regions and an excess of duplicated sequences in homozygous regions, although the effect on genome size inflation was weaker than in *C. melanoneura*. Although the genome size and repetitiveness seem to be properties inherent to both genomes, the heterozygosity and efficiency in collapsing haplotypes can be explained by the larger number of individuals sequenced and simultaneously assembled in *C. melanoneura*.

### Mitochondrial genomes of *C. melanoneura* and *C. picta*

The final assemblies of the *C. melanoneura* and *C. picta* mitogenomes yielded 14,882 bp and 14,829 bp circular sequences, respectively, with 13 PCGs, 22 tRNAs and 2 rRNAs, as well as some control region and origin of L-strand replication elements (recovered in *MITOS2*) (Fig. S2). The mitochondrial genome size of *C. melanoneura* presented here was almost identical to the one of specimens from Czech Republic (14,779 − 14,881 bp) [27], while the *C. picta* specimen of this study had a slightly larger mitochondrial genome compared to Czech individuals (14,801 and 14,802 bp) [27]. In general, variations in genome length among the investigated *Cacopsylla* species are consistently found in the control regions. The gene order and GC usage in both *C. melanoneura* and *C. picta* was identical to that observed across all known *Cacopsylla* species [26, 69–71] as well as most other psyllid species [26, 27].

### Mitochondrial phylogeny and divergence time estimation of *Cacopsylla* species

We investigated the phylogenetic relationships of the newly sequenced samples of *C. melanoneura* and *C. picta* within the genus *Cacopsylla*. Our dataset comprised 13 PCGs obtained from whole-mitochondrial sequences across 12 *Cacopsylla* species and 12 outgroup taxa (Table 1 and Table S6). To assess the robustness of our phylogenetic inference, we employed two analytical frameworks, Bayesian inference (BI) and maximum likelihood (ML), and analysed three data types: amino acid sequences (3,404 bp), nucleotide sequences including all codon positions (10,211 bp), and nucleotide sequences with the third codon position excluded, hereafter referred to as the nucleotide tree (6,809 bp).

**Table 1:** Biological traits, vector status, host associations and geographic distribution of 12 *Cacopsylla* species included in the phylogenetic and transposable elements analyses

Voltinism, the number of generations per year; Overwintering strategy, the developmental stage and plant on which psyllid overwinters; Dimorphism, the presence of seasonally distinct morphological forms; Vector status denotes whether the species is a confirmed vector of plant pathogens (*Liberibacter, Phytoplasma*); Host plant species/family, the plant taxa on which psyllid completes its life cycle; Distribution, the known geographic range of each species

For the time-calibrated BI phylogenies, we assessed the fit of different tree priors by comparing marginal likelihood estimates (MLE) derived via stepping-stone sampling. The MLE values for the Yule and birth–death models were − 106,096.04 and − 106,088.62, respectively. The resulting Bayes Factor (BF = -7.42) favoured the birth–death model, which we therefore used in the final analyses.

**Fig. 1:**
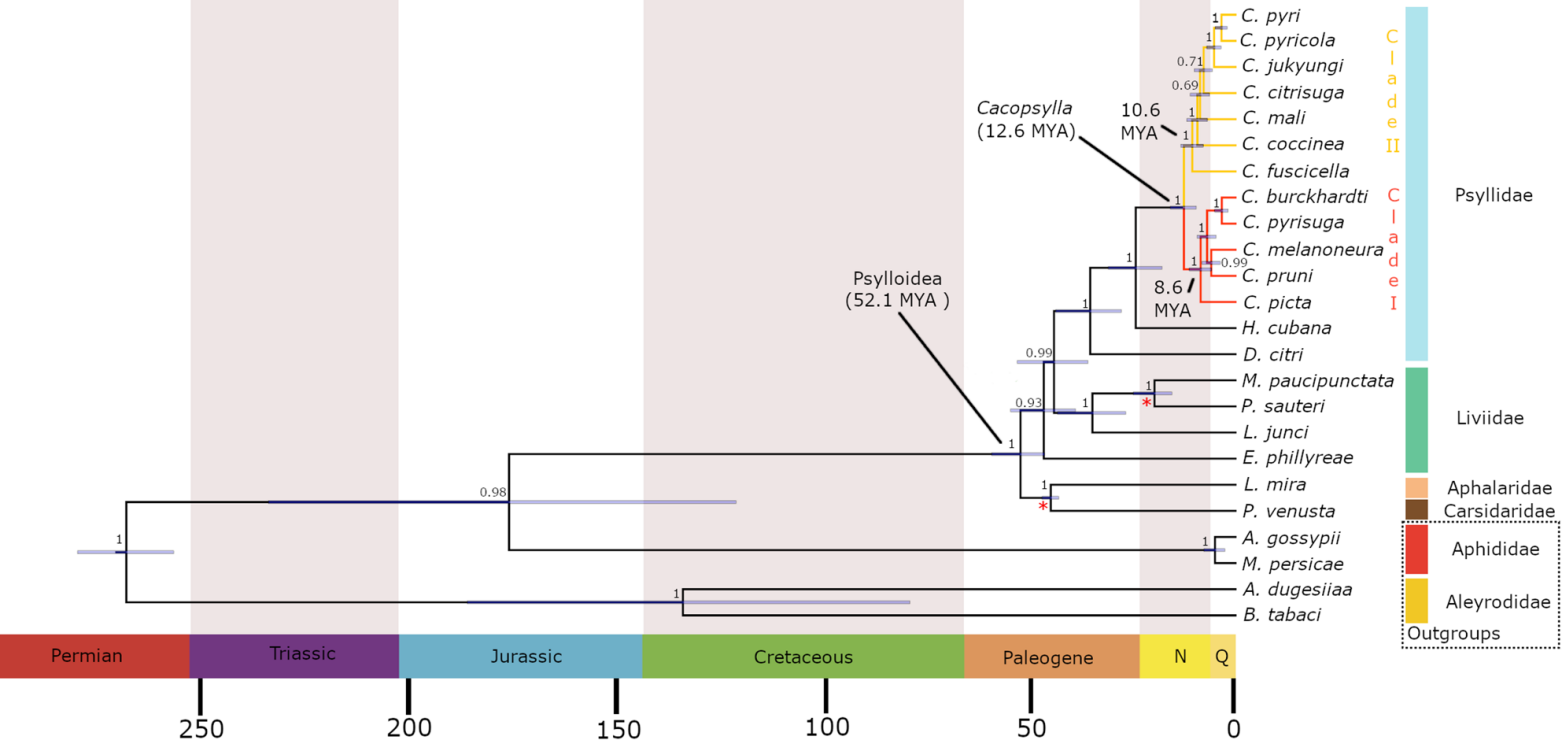
Time-calibrated Bayesian phylogeny of 12 *Cacopsylla* species, based on 13 mitochondrial PCGs. Red branches correspond to Clade I and yellow branches to Clade II. The * refers to selected fossil records used for calibrations. Number at nodes correspond to posterior probabilities derived from the amino acid dataset. Node divergence age estimates ± 95% HPD are shown to be overlapping based on nucleotide third codon removed phylogeny

In general, the BI trees based on the amino acid dataset and the nucleotide dataset excluding the third codon position, provided stronger support for both deep and internal branches suggesting that these values provide more reliable approximate estimates compared to the all-codon tree (Fig. 1; S3, S4, S5). A notable difference in the all-codon tree, however, was the recovery of the outgroup family Liviidae as monophyletic, including *Euphyllura phillyreae* (Euphyllurinae) and three representatives of the subfamily Liviinae (*Livia junci*, *Melanastera paucipunctata*, *Paurocephala sauteri*). In addition, the all-codon tree recovered a monophyletic grouping of *C. coccinea* and *C. mali* (Fig. S3).

**Fig. 2:**
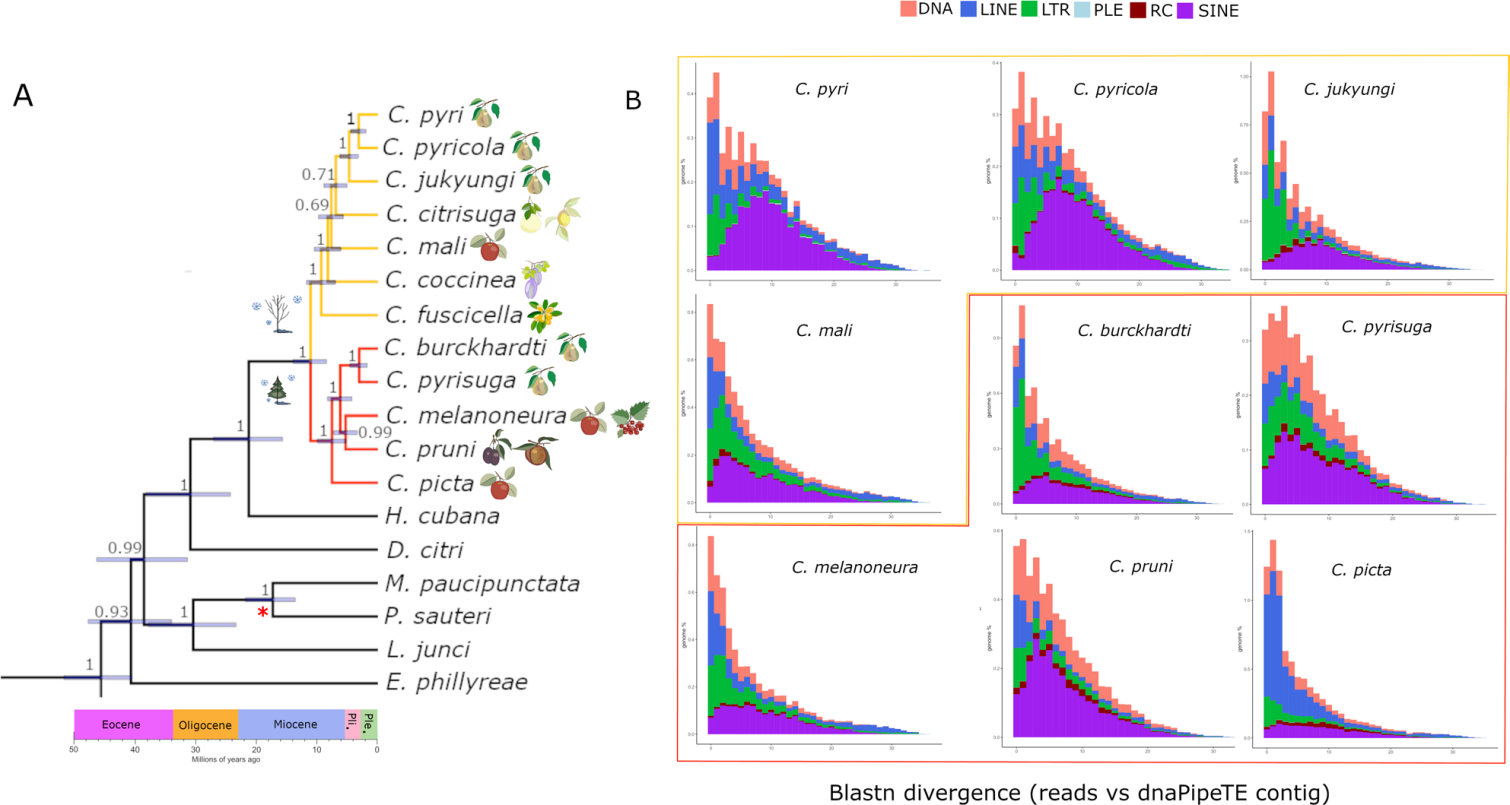
Subtree with dated phylogeny of *Cacopsylla* species with repeat landscape plots. **A** Subtree from Fig. 1. Icons of the leafless host plant tree and the conifer indicate differences in overwintering behaviour (migrating - in red (Clade I); non-migrating - in yellow (Clade II), whereas icons of different fruits represent the most agriculturally important host plants for each species. **B** Landscapes divergence in TEs, the X-axis of each plot reflects how much a TE has diverged from its consensus sequence, with low divergence suggesting younger TEs (peaks on the left), while high divergence indicates ancient insertions (broad right-shifted distributions). The Y-axis represents the genomic proportion of the genome occupied by TEs at each divergence level. The large fraction of unclassified elements was omitted for these plots to focus only on annotated elements

The divergence time estimates from the amino acid tree were in general older compared to the nucleotide tree. In both trees, the crown diversification of Psylloidea was estimated to have occurred in the Paleocene–Eocene, at approximately 52.1 MYA (95% HPD: 45.7–70.2 MYA) based on the nucleotide dataset, or 62.3 MYA (95% HPD: 46.3–82.7 MYA) based on the amino acid dataset (Fig. 1). These estimates are substantially older than the fossil prior minimum of 41.3 MYA. In the amino acid tree, the crown age of the genus *Cacopsylla* was estimated to be in the Early Miocene, at approximately (95% HPD: 10.8–27.5 MYA). In contrast, the nucleotide tree suggested a later divergence, placing the crown age of *Cacopsylla* in the Middle Miocene, at approximately 12.7 MYA (95% HPD: 9.7–16.0 MYA). Both the amino acid and nucleotide trees strongly supported (PP = 1.0) the presence of two major *Cacopsylla* clades: Clade I and Clade II (Figs. 1 and 2). All species within *Cacopsylla* Clade I (red branches) are associated with host plants from the family Rosaceae (Table 1; Fig. 2). In all trees, *C. melanoneura* forms a clade with the plum psyllid *C. pruni*, with strong support in the amino acid tree (PP = 0.96) and in the nucleotide tree (PP = 1.0). The divergence between these two species was estimated at approximately 6.1–6.9 MYA (amino acid tree: 95% HPD: 2.4–11.8 MYA; nucleotide tree: 95% HPD: 3.9–8.3 MYA). This pair consistently clusters as a sister group to the pear psyllids *C. burckhardti* and *C. pyrisuga*, with strong support across both datasets (amino acid tree PP = 1.0; nucleotide tree PP = 0.99). In turn, *C. picta* forms a sister lineage to the above-mentioned clades in both trees (PP = 1.0), with the split between *C. picta* and the most recent common ancestor (MRCA) of these species estimated at approximately 8.6–11.5 MYA, in the Late Miocene (amino acid tree: 95% HPD: 5.5–18.7 MYA; nucleotide tree: 95% HPD: 6.0–11.4 MYA). These results suggest independent evolutionary trajectories for the two main AP vector species in Europe, *C. melanoneura* and *C. picta*.

The *Cacopsylla* species in Clade II (yellow branches) form a separate group, also including psyllids primarily associated with Rosaceae, along with single representatives from the families Lardizabalaceae and Rutaceae (Table 1). The two European species associated with *Pyrus*, *C. pyri* and *C. pyricola*, cluster together with the Asian *C. jukyungi*, also associated with *Pyrus*, as sister species (Fig. 2). These nodes exhibit strong support across all phylogenetic inference methods. In contrast, the phylogenetic relationships among *C. citrisuga*, *C. coccinea*, and *C. mali* remain unresolved and vary considerably among different phylogenetic reconstructions, likely due to incomplete taxon sampling. Interestingly, *C. citrisuga* and *C. coccinea* are Asian species that primarily reproduce on citrus and *Akebia*, respectively, while *C. mali*, found in Europe and North America, reproduces on apple trees. Notably, although it is the only species in this group associated with apple, *C. mali* is phylogenetically distant from *C. melanoneura* and *C. picta* and has never been recovered as their sister taxon.

Another notable finding is that the divergence of the four pear-associated *Cacopsylla* species from the two clades, *C. pyri* / *C. pyricola* (Clade II) and *C. burckhardti* / *C. pyrisuga* (Clade I), represents the two most recent speciation events among the analysed taxa, estimated at around 4.7 MYA (amino acid tree: 95% HPD: 3.6–6.1 MYA; nucleotide tree: 95% HPD: 4.6–6.1 MYA). Similar to the results of the BI all-codon tree, the ML all-codon tree also recovered the family Liviidae as monophyletic, albeit with weak support (Fig. S6). In contrast, both the BI and ML trees based on amino acid and third codon-excluded nucleotide datasets did not support the monophyly of Liviidae, while both major *Cacopsylla* clades (Fig. S7 and S8) were strongly supported in all ML analyses (BS > 0.98), with internal branch support across *Cacopsylla* being generally lower than in the BI inferences.

### Repetitive element landscape diversity in *Cacopsylla* species

TE composition was investigated using landscape plots that report the frequency and variability of the different TE families. In this analysis, sequence divergence of TE copies from their consensus sequence is shown on the X-axis, and their genomic proportion on the Y-axis. Peaks toward the left (low divergence) indicate more recent TE insertions, while those toward the right (high divergence) reflect older insertions. In *C. mali* and *C. picta*, prominent recent bursts of DNA and LINE elements support relatively recent TE bursts. In contrast, species like *C. pruni* and *C. pyricola* show older TEs expansions (Fig. 2). The dominant TE classes vary across samples, indicating that different TE families drive genome changes in different species (Fig. 2). Specifically, in *C. pyri*, there is high recent TE activity, particularly in DNA and LINE elements, while in its sister species, *C. pyricola*, recent TE activity is lower and the TE landscape suggests older insertions. *Cacopsylla jukyungi* shows DNA and LTR elements dominant in recent bins, suggesting a likely recent expansion. In *C. mali*, there is a very pronounced peak of low divergent TE copies, supporting strong recent activity of DNA, LINE and RC elements. This is likely one of the highest recent TE activity profiles. In *C. burckhardti*, there is moderate to high recent TE activity, mostly driven by LTR, DNA and LINE elements. The sister species, *C. pyrisuga*, has a flatter and more right-shifted TE distribution, which suggests that TE activity has slowed down. *Cacopsylla melanoneura* shows very active recent TE insertions, especially in DNA, LINE and LTR elements. The prominent peak at 0–2% divergence suggests a recent expansion event. In contrast, the sister species *C. pruni* shows low recent TE activity, with most insertions being older. The TE peak is broad and right shifted, indicating a more stable TE landscape. Finally, *C. picta* has a sharp recent peak, especially in LINE and DNA elements, which is comparable to *C. mali* and *C. melanoneura*, making it one of the most dynamic genomes in this dataset. In summary, the species that show strong peaks at very low divergence values (0–2%), indicating ongoing or recent TE bursts, are *C. mali* from Clade II and all the species from Clade I, except *C. pruni*. The remaining species from Clade II and *C. pruni* show a broader peak indicating relatively low and moderate divergence values (3–10%).

### TE content contributes to genome size in *C. melanoneura* and *C. picta*

Given the substantial difference in estimated genome size between *C. melanoneura* and *C. picta*, we examined in more detail the contribution of repetitive elements to their genome size. Repetitive elements accounted for a moderate fraction of the assembled genome of both *C. melanoneura*, at 6.7%, and *C. picta*, at 12.2%. In both species, simple repeats were the most common element, followed by retroelements (LINEs, SINEs and LTRs), and DNA transposons (Fig. S9). This enrichment was consistent when considering total element count, length, and percentage of genome occupied. Focusing on comparative differences between species, simple repeats were more numerous and accounted for a larger proportion of the genome in *C. picta* compared to *C. melanoneura* (Fig. S9). A similar pattern was observed for retroelements, which was driven by the relative abundances of LINE elements (Fig. S9). Unclassified repetitive elements were also more common in *C. picta*. No notable differences in counts, length or percentage of genome occupied were found for SINE or LTR retroelements, sRNA or satellites, while DNA transposons were slightly more numerous in *C. picta*, although the percentage of genome occupied by these TEs was identical in the two species. Similar results were obtained using *RepeatMasker* with species-specific repeat libraries generated by *RepeatModeler2*, where the main difference observed was in the proportion of masked bases, 52.95% in *C. melanoneura* and 60.86% in *C. picta.* Discrepancies between the two approaches were primarily due to differences in the proportion of unclassified repetitive elements in both species (Fig. S9).

### Retroelements and DNA transposons in the draft genome of *C. melanoneura* and *C. picta*

We further explored the genomic abundance in the draft genome assembly of both the retroelement and DNA transposon sub-classes. LINEs were the most abundant subclass in both *C. melanoneura* and *C. picta*, followed by SINEs and LTRs (Fig. S9; S10). However, LINEs were more abundant in *C. picta*, while SINEs and LTRs were more common in *C. melanoneura*. Most of the difference in LINE content between the two species is associated to the much higher number of *RTE/Bov-B* elements in *C. picta*. On the other hand, the higher abundance of LTRs in *C. melanoneura* is mostly attributed to BEL/Pao and *Gypsy/DIRS1* being more abundant in this species (Fig. S10). We also note that *Tcl-IS630-Pogo* and *hobo-activator* were the most common DNA transposons in both *C. melanoneura* and *C. picta*, the latter hosting in particular almost twice as much *hobo-activator* than its related species (Fig. S10).

## Discussion

Psyllids from the genus *Cacopsylla* are phytophagous insects with a wide range of host plants and include several vectors of plant pathogens. Although several *Cacopsylla* species are among the most important pests of various fruit trees, information on their evolutionary history, including their genetic and genomic makeup and evolution, remains scarce. We therefore conducted comparative genomic and phylogenetic analyses of *Cacopsylla* species, with a special focus on the two vectors of apple phytoplasma, ‘*Ca.* P. mali’: *C. melanoneura* and *C. picta*. One specific aim was to assess whether their ability to transmit ‘*Ca.* P. mali’ reflects a shared evolutionary origin or independent acquisition. For this purpose, we sequenced and assembled the nuclear and mitochondrial genomes of these species and, leveraging on publicly available data from other *Cacopsylla* and other psyllid species, reconstructed their phylogeny to trace their relationships with other vectors within the genus *Cacopsylla*.

The phylogenetic trees based on amino acid and nucleotide sequences of 12 psyllid species showed largely similar topologies, although divergence times were consistently estimated to be older when inferred from the amino acid data. In both, the amino acid and nucleotide phylogenies, *Euphyllura phillyreae* from the subfamily Euphyllurinae was recovered as paraphyletic with respect to the other analysed Liviinae representatives. In contrast, the family Liviidae was recovered as monophyletic in the all-codon trees. A similar pattern was reported by Percy et al. [26], who found that Liviidae is not monophyletic in either their conserved codon or combined datasets. Nevertheless, our amino acid and nucleotide phylogenies recovered stronger support for both deep and internal clades compared to the all-codon trees, suggesting that codon composition and substitution saturation may affect the inferred topology. This contrasts with findings from Wang et al. [72], where excluding the third codon position from nuclear nucleotide datasets did not resolve compositional heterogeneity or correct the inferred topology. We estimated that Psylloidea started diversifying during the Paleocene–Eocene, approximately at 52.1 or 62.3 million years ago (MYA), with confidence intervals ranging from 46.3 to 82.7 MYA. These estimates are substantially younger than those reported in the large-scale Hemiptera phylogenies of Li et al. [63] that placed the crown diversification of Psylloidea at ~ 120 MYA during the Early Cretaceous, while Johnson et al. [73] estimated it at ~ 220 MYA in the Late Triassic. This discrepancy may be explained by our inclusion of psyllid fossil calibrations, which constrained internal psyllid nodes, as well as our broader taxon sampling within Psylloidea. Our results are also consistent with paleontological evidence presented by Ouvrard [64] and Drohojowska et al. [65, 74], which suggested that, based on fossil evidence, extant psyllids could not have diversified earlier than the mid-Eocene Lutetian stage. Although our point estimates predate the Lutetian, the lower bound of the 95% HPD interval extends into this stage, making our divergence estimates more consistent with both paleontological and molecular evidence than previous studies.

The crown divergence of the genus *Cacopsylla* was estimated to have occurred in the Early Miocene at 18.4 MYA (amino acid phylogeny) or in the Middle Miocene at 12.7 MYA (nucleotide phylogeny). These estimates are also in agreement with the fossil evidence of *Cacopsylla* dated to the Miocene [75]. Because the upper and lower bounds of the amino acid and nucleotide-based estimates also overlap, the approximate dating is reliable. Nevertheless, the amino acid or nucleotide-based estimate is probably the closest estimate of the true time of divergence given its better alignment with the available fossil evidence. This time estimation, based on 13 mitochondrial protein coding genes and psyllid fossil calibrations, contrasts with the results of a recent study by Darnis et al. [76], who estimated the crown divergence of *Cacopsylla* at approximately 85 MYA. Their analysis was based on a 621 bp fragment of the Cytochrome c oxidase subunit I–tRNA–COII region, combined with limited fossil evidence and external calibration derived from the phylogeny of Rosaceae. The discrepancy between the two studies may be attributed to the use of a single gene in the latter study as well as differences in calibration methods.

Our topologies across all trees consistently support a close relationship between *C. melanoneura* and *C. pruni*, the latter being the major vector of ‘*Ca.* Phytoplasma prunorum’, the causal agent of European Stone Fruit Yellows [77]. Interestingly, both *C. melanoneura* and *C. pruni* may represent cryptic species complexes, comprising genetically distinct lineages that are morphologically nearly identical and lack diagnostic morphological characters to distinguish them. These lineages are generally associated with different host preferences and show considerable molecular divergence within each nominal species [24, 28, 76, 78, 79].

Crucially, across nearly all analyses, *C. picta* was consistently placed as a sister taxon to a clade comprising four other Rosaceae-associated species, *C. melanoneura* and *C. pruni*, as well as two pear psyllids *C. burckhardti* and *C. pyrisuga*. This pattern suggests that the two primary vectors of AP, *C. melanoneura* and *C. picta*, likely evolved their vector competence independently rather than inheriting it from a common ancestor. Furthermore, both AP vector species are even more distantly related to another apple psyllid *C. mali*, which has not been confirmed as a vector of ‘*Ca.* P. mali’. Similarly, pear psyllids *C. pyri* and *C. pyricola* (Clade II), as well as *C. burckhardti* and *C. pyrisuga* (Clade I), are distributed across distinct phylogenetic clades, suggesting independent evolutionary origins of pear-associated lineages. While *C. pyri* and *C. pyricola* may have inherited vector competence from a common ancestor, the more distantly related *C. pyrisuga* likely acquired this trait through a separate evolutionary pathway.

While *Cacopsylla* species do not cluster based on their capability to transmit phytoplasma or host preference, the two clades recovered in our study appear to differ in key biological traits. *Cacopsylla* Clade I consists of species which are strictly associated with Rosaceae. All species in this clade ((*C. burckhardti* + *C. pyrisuga*), (*C. melanoneura* + *C. pruni*), *C. picta*) are univoltine and migrate to conifers for overwintering (Table 1). This migratory behaviour may represent an adaptation to harsh environmental conditions and the seasonal unavailability of suitable phloem sources on deciduous hosts, serving as an overwintering strategy of many psyllid species in Northern temperate regions [23]. In contrast, most species assigned to Clade II (*C. pyri*, *C. pyricola*, *C. jukyungi*, *C. citrisuga*, *C. mali*) overwinter on their host plants without migrating to conifers. Although the genetic basis and evolution of the migratory behaviour in psyllids remain unclear, our data suggest a potential correlation between phylogenetic structure and ecological traits. Moreover, all species belonging to Clade I are restricted to the family Rosaceae, whereas several species from Clade II are associated with other plant families, i.e. *C. citrisuga* develops on Rutaceae and *C. coccinea* on Lardizabalaceae. Additionally, Clade I contains only univoltine species, while various species belonging to Clade II are polyvoltine. This pattern is consistent with ecological strategies observed in psyllids from tropical and Southern temperate regions, where the psyllid species richness is the highest and continuous host availability facilitates multiple generations per year [1]. Taken together, these observations suggest that life history traits, such as voltinism and overwintering strategy, might be linked to the phylogenetic structure within *Cacopsylla*, and may help trace the evolutionary transitions in host association and overwintering behaviour. However, broader taxon sampling and mitogenome sequencing across the genus are needed to confirm this hypothesis.

We also investigated the repeatome to better understand the genomic processes shaping evolution in the *Cacopsylla* genus. We found that TEs dynamics vary widely across species, with lineage-specific patterns of accumulation and turnover likely contributing to genome evolution and adaptation. Species such as *C. mali*, *C. melanoneura*, *C. picta* and *C. pyri* show evidence of recent TE bursts, particularly of DNA transposons and LINE elements, suggesting ongoing genome re-modelling. In contrast, *C. pruni*, *C. pyricola*, and *C. pyrisuga* exhibit older and likely more stable TE landscapes. According to our phylogenetic analyses, Clade I and Clade II are similar in age, although Clade I may be slightly younger. Moreover, the two clades differ in life history traits that may influence TE activity (Table 1). Clade I species inhabit less stable environments and are univoltine and migratory, with seasonal movement to conifers for overwintering. In contrast, most Clade II species are mostly polyvoltine, with several of them occupying more stable environments (without seasonal changes), which may reduce the stressful conditions that promote TE activity [80–82]. Environment aside, polyvoltine species may accumulate TEs faster due to shorter generation times [83], while univoltine species may experience slower TE accumulation and stronger selection against harmful insertions and more stable epigenetic silencing [84]. Additionally, the presence of seasonal migration in some species may also lead to periods of genomic “silence” potentially affecting TE activity compared to species that undergo continuous generations on their host plants. For example, studies have shown that in some insect groups, TEs are differentially expressed during diapause versus active developmental stages. In the bee species *Megachile rotundata* and *Osmia lignaria*, TE expression varied significantly during diapause and in response to temperature stress, suggesting that environmental conditions and life stage transitions can modulate TE activity [85]. Similarly, in *Drosophila montana*, a cold-adapted fly species, specific TEs were found to be upregulated during diapause, implying a role in regulating genes associated with diapause and cold tolerance [86].

We further compared the TEs in the shallow genome assemblies of *C. melanoneura* and *C. picta*. The primary finding was that simple repeats, LINE-class retrotransposons, and unclassified repeats were approximately twice as abundant in the genome of *C. picta* compared to *C. melanoneura*. The analysis of retroelements and DNA transposons revealed that these classes significantly contributed to the genome size differences between the two species. Retroelements such as RTE/Bov-B subclass were especially overrepresented in *C. picta*. In contrast, *C. melanoneura* had a greater proportion of non-assigned LINEs, although this did not affect the overall retroelement composition. Overall, while the composition of element types was broadly conserved, *C. picta* consistently exhibited a higher abundance of most repeat types.

Transposable elements may contribute to genomic variability and regulatory plasticity that may facilitate resistance to chemical contaminants such as pesticides, primarily through gene upregulation, loss of function, and alternative splicing. Among TE types, LINEs have been shown in *Drosophila melanogaster* to alter the splicing and regulation of genes involved in insecticide resistance, conferring resistance to DDT and other insecticides [87, 88]. Similarly, in *Helicoverpa armigera*, several TE families, including LINEs, have been identified near detoxification genes, supporting the association between TE activity and gene regulation [89, 90]. Similarly, in the whitefly *Bemisia tabaci*, multiple TE insertions were reported, including six linked to known insecticide resistance genes, suggesting a role in both resistance and genome evolution [91]. In the aphid *Myzus persicae*, TEs contribute to resistance by disrupting a dominant susceptibility allele, enabling the expression of a recessive resistance allele [92]. These studies demonstrate how TEs can drive adaptation under environmental and genetic stressors highlighting a potential role of TEs in the adaptation of agriculturally important pest species such as *C. picta* and *C. melanoneura* on pesticides.

## Conclusions

*Cacopsylla* is a diverse genus of phytophagous insects which includes several important vectors of phytoplasma, such as Apple Proliferation (AP), a serious threat to agriculture. Using a time-calibrated phylogenetic approach, we estimated the crown divergence of this genus between the Early Miocene (~ 18.4 MYA) and the Middle Miocene (~ 12.7 MYA). Within *Cacopsylla*, two major clades were identified, largely differentiated by their overwintering strategies and voltinism, ecological traits that might reflect adaptations to environmental changes over the past 10 million years. Interestingly, although *C. melanoneura* and *C. picta* are the only *Cacopsylla* species known to be vector of ‘*Ca.* P. mali’ and both develop on apple trees, we found them to be phylogenetically distantly related. The divergence of *C. picta* from the most recent common ancestor of *C. melanoneura* was estimated to have occurred between 8.6 and 11.5 MYA, during the Late Miocene. These findings reinforce the hypothesis that these two species vectoring AP phytoplasma followed independent evolutionary trajectories and likely acquired the ability to transmit ‘*Ca.* P. mali’ independently. Furthermore, *C. mali*, another apple-associated psyllid that is not a confirmed vector of ‘*Ca.* P. mali’, was found to belong to a distinct clade and was never recovered as a sister taxon to either *C. melanoneura* or *C. picta*. Based on the current results, it is not possible to clearly identify the ancestral clade, although Clade I appears relatively younger than Clade II. Finally, our analyses of transposable elements suggest that life history traits, such as host preference, overwintering strategy and voltinism, may influence recent genome rearrangements. These patterns are more pronounced in the representatives from the generally univoltine and overwintering Clade I, supporting the idea that some repetitive elements may play functional roles in seasonal migration, ecological adaptation, pathogen transmission and potentially in the evolution of pesticide resistance. Future studies are needed to clarify how transposable element dynamics, and the expansion of repetitive sequences shape genomic plasticity and resilience in these agriculturally important pests.

## Supplementary Information

Supplementary Material 1.

## Data Availability

NCBI BIO PROJECT: PRJNA1312285.

## Abbreviations

AP: Apple Proliferation
BF: Bayes Factor
BI: Bayesian Inference
BS: Bootstrap
COI: Cytochrome c Oxidase Subunit I
ESS: Effective Sampling Size
HPD: Highest Posterior Density
LINEs: Long Interspersed Nuclear Elements
LTRs: Long Terminal Repeats
MCC: Maximum Clade Credibility
ML: Maximum Likelihood
MLE: Marginal Likelihood Estimation
MRCA: Most Recent Common Ancestor
MYA: Million Years Ago
PCGs: Protein-Coding Genes
PP: Posterior Probabilities
RC: Rolling Circle Transposon
SINEs: Short Interspersed Nuclear Elements
TEs: Transposable Elements
