# Supplementary material for: Evolutionary genomics and divergence of Cacopsylla species with a special focus on the apple proliferation vectors Cacopsylla melanoneura and Cacopsylla picta
Source: BMC Genomics. 2026 Mar 19;27:285. doi: 10.1186/s12864-026-12622-0 (PMC13003732; doi:10.1186/s12864-026-12622-0)
Supplement: Supplementary file 1 — Supplementary Material 1. [file 12864_2026_12622_MOESM1_ESM.docx]

Supporting information: Evolutionary genomics and divergence of *Cacopsylla* species with a special focus on the Apple Proliferation Vectors: *Cacopsylla melanoneura* and *C. picta*

Lapo Ragionieri^1¥*^, Liliya Štarhová Serbina^2,3¥^, Erika Corretto^1^, James M. Howie^4,5^, Fernando Cruz ^6,7^, Tyler S. Alioto^6,7^, Nicola Zadra^8^, Tobias Weil^9^, Gianfranco Anfora^8^, Christian Stauffer^4^, Lino Ometto^10*^, Omar Rota-Stabelli,^8,9*^, Hannes Schuler^1,11^

^1^ *Competence Centre for Plant Health, Free University of Bozen-Bolzano, Bozen-Bolzano, Italy*

^2^ *Center for Integrative Biodiversity Discovery, Leibniz Institute for Evolution and Biodiversity Science; Museum für Naturkunde, Berlin, Germany*

^3^ *Present address: Museum of Nature Hamburg, Leibniz Institute for the Analysis of Biodiversity Change, Hamburg, Germany*

^4^ *Department of Ecosystem Management, Climate and Biodiversity, BOKU University, Vienna, Austria*

^5^ *Present address: Medical University of Vienna, Center for Cancer Research, Vienna, Austria*

^6^ *Centro Nacional de Análisis Genómico (CNAG), Baldiri Reixac 4, 08028 Barcelona, Spain*

*^7^ Universitat de Barcelona (UB), Barcelona, Spain*

^8^ *University of Trento, Center Agriculture Food Environment (C3A), Trento, Italy*

^9^ *Centro Ricerca ed Innovazione, Fondazione Edmund Mach, San Michele all’ Adige,* Italy

^10^ *Department of Biology and Biotechnology, University of Pavia, Pavia, Italy*

^11^ *Free University of Bozen-Bolzano, Faculty of Natural Sciences and Technology, 39100, Bozen, Italy*

^*^Authors for Correspondence, ^¥^Contributed equally

**Table S1.** Sequencing data of *Cacopsylla melanoneura* and *Cacopsylla picta*

| **Sample**  **name** | **Library** | **Read length (bp)** | | **Fragment length (bp)**^1^ | | | **Yield (Gb)** | | **Coverage**^2^ | | **phix error r1 (%)** | | **phix error r2 (%)** |
| --- | --- | --- | --- | --- | --- | --- | --- | --- | --- | --- | --- | --- | --- |
| Cmel_1 | PE310 | 2x251 | | 310 | | | 57.85 | | 131.87 | | 1.745 | | 1.845 |
| Cmel_2 | PE290 | 2x251 | | 289 | | | 59.37 | | 135.34 | | 1.745 | | 1.845 |
| Cmel_4 | PE700 | 2x251 | | 689 | | | 75.34 | | 171.74 | | 1.5725 | | 1.9525 |
| Cpic_2 | PE510 | | 2x251 | | 507 | 121.46 | | 192.15 | | 1.5725 | | 1.953 | |
| Cpic_2 | PE750 | | 2x251 | | 746 | 74.94 | | 118.57 | | 1.5725 | | 1.953 | |

^1^ Fragment size used during agarose gel size selection for *C. melanoneura*. Paired End Libraries are referred as PE310, PE290 and PE700 attending to their genomic fragment size. ^2^ The sequencing coverage was calculated assuming a genome size of 438.7 Mb (as it was estimated with Genomescope v1.1 using a k=17).

^1^ Fragment size used during agarose gel size selection (including adapter and other technical sequences) for *C. picta*. Paired End Libraries were named PE510 and PE750 attending to their genomic fragment size. ^2^ The sequencing coverage was calculated assuming a genome size of 632.08 Mb (as it was estimated with Genomescope v1.1 using a k=17).

**Table S2.** Genome Properties of *Cacopsylla melanoneura* and *Cacopsylla picta*

|  | **Property*** | **min** | **max** |
| --- | --- | --- | --- |
| ***Cacopsylla melanoneura*** | Heterozygosity | 2.51% | 2.53% |
|  | Genome Haploid Length (bp) | 438,045,262 | 438,672,560 |
|  | Genome Repeat Length (bp) | 250,936,758 | 251,296,110 |
|  | Genome Unique Length (bp) | 187,108,503 | 187,376,450 |
|  | Model Fit | 86.89% | 94.21% |
|  | Read Error Rate | 1.05% | 1.05% |
|  | Repetitive Fraction of the Genome | 57.28% | 57.28% |
| ***Cacopsylla picta*** | Heterozygosity | 1.37% | 1.37% |
|  | Genome Haploid Length (bp) | 631,347,990 | 632,082,702 |
|  | Genome Repeat Length (bp) | 423,571,562 | 424,064,480 |
|  | Genome Unique Length (bp) | 207,776,428 | 208,018,222 |
|  | Model Fit | 85.59% | 97.20% |
|  | Read Error Rate | 0.68% | 0.68% |
|  | Repetitive Fraction of the Genome | 67.09% | 67.09% |

^*^Estimated from the Illumina PE700 2x251bp reads using GenomeScope version 1.0 (k = 17)

**Table S3.** Contaminant Species detected with Kraken

|  | **Species Name** | **NCBI TaxID** | **%Reads Covered** |
| --- | --- | --- | --- |
| ***Cacopsylla melanoneura*** | *Myceliophthora thermophila* | 78579 | 1.63 |
|  | *Candida dubliniensis* | 42374 | 1.26 |
|  | *Leishmania major* | 5664 | 1.14 |
|  | *Thielavia terrestris* | 35720 | 0.46 |
|  | *Methanobacterium formicicum* | 2162 | 0.31 |
|  | *Streptococcus sp. VT 162* | 1419814 | 0.26 |
|  | *Saccharomyces cerevisiae* | 4932 | 0.18 |
|  | *Pandoravirus dulcis* | 1349409 | 0.08 |
|  | *Cronobacter sakazakii* | 28141 | 0.07 |
|  | *Alteromonas mediterranea* | 314275 | 0.06 |
|  | *Piscine myocarditis-like virus* | 1798085 | 0.06 |
|  | *Mycoplasma hyopneumoniae* | 2099 | 0.06 |
|  | *Kluyveromyces lactis* | 28985 | 0.05 |
|  | *Methanococcus voltae* | 2188 | 0.04 |
|  | *Candidatus Carsonella ruddii* | 114186 | 0.03 |
|  | *Pandoravirus salinus* | 1349410 | 0.03 |
|  | *Methanosarcina lacustris* | 170861 | 0.03 |
| ***Cacopsylla picta*** | *Bacillus halodurans* | 86665 | 4.31 |
|  | *Methanobacterium formicicum* | 2162 | 4.24 |
|  | *Myceliophthora thermophila* | 78579 | 2.57 |
|  | *Candida dubliniensis* | 42374 | 2.35 |
|  | *Leishmania major* | 5664 | 1.45 |
|  | *Thielavia terrestris* | 35720 | 0.79 |
|  | *Alteromonas mediterranea* | 314275 | 0.58 |
|  | *Candidatus Carsonella ruddii* | 114186 | 0.57 |
|  | *Saccharomyces cerevisiae* | 4932 | 0.37 |
|  | *Streptococcus sp. VT 162* | 1419814 | 0.26 |
|  | *Buchnera aphidicola* | 9 | 0.13 |
|  | *Methanococcus voltae* | 2188 | 0.12 |
|  | *Mycoplasma hyopneumoniae* | 2099 | 0.11 |
|  | *Piscine myocarditis-like virus* | 1798085 | 0.1 |
|  | *Kluyveromyces lactis* | 28985 | 0.1 |
|  | *Pandoravirus dulcis* | 1349409 | 0.09 |
|  | *Methanosarcina lacustris* | 170861 | 0.05 |

**Table S4.** Summary contiguity statistics of *Cmel*1 assembly

|  | **Contigs** | | **Scaffolds** | |
| --- | --- | --- | --- | --- |
|  | **Length (bp)** | **Number** | **Length (bp)** | **Number** |
| **N0** | 23,792 | 1 | 94,132 | 1 |
| **N5** | 4,910 | 5,378 | 16,600 | 1,634 |
| **N10** | 3,867 | 13,235 | 13,042 | 3,999 |
| **N15** | 3,280 | 22,779 | 10,934 | 6,900 |
| **N20** | 2,860 | 33,846 | 9,409 | 10,303 |
| **N25** | 2,542 | 46,411 | 8,168 | 14,234 |
| **N30** | 2,279 | 60,472 | 7,108 | 18,755 |
| **N35** | 2,056 | 76,100 | 6,182 | 23,956 |
| **N40** | 1,866 | 93,375 | 5,361 | 29,939 |
| **N45** | 1,694 | 112,403 | 4,612 | 36,869 |
| **N50** | **1,539** | 133,352 | **3,921** | 44,968 |
| **N55** | 1,398 | 156,417 | 3,254 | 54,610 |
| **N60** | 1,266 | 181,840 | 2,624 | 66,389 |
| **N65** | 1,135 | 209,999 | 2,040 | 81,300 |
| **N70** | 997 | 241,779 | 1,572 | 100,578 |
| **N75** | 856 | 278,276 | 1,200 | 125,681 |
| **N80** | 688 | 322,177 | 940 | 158,244 |
| **N85** | 523 | 378,576 | 650 | 201,835 |
| **N90** | 398 | 454,011 | 432 | 267,199 |
| **N95** | 303 | 550,515 | 317 | 359,291 |
| **N100** | 51 | 678,097 | 250 | 483,574 |
| **Total** | 676,207,714 | 678,097 | 688,997,636 | 483,574 |

**Table S5.** Summary contiguity statistics of *Cpic*2 assembly

|  | **Contigs** | | **Scaffolds** | |
| --- | --- | --- | --- | --- |
|  | **Length (bp)** | **Number** | **Length (bp)** | **Number** |
| **N0** | 60,181 | 1 | 83,674 | 1 |
| **N5** | 7,692 | 3,578 | 24,248 | 1,184 |
| **N10** | 5,868 | 9,127 | 18,815 | 2,937 |
| **N15** | 4,917 | 16,006 | 15,654 | 5,102 |
| **N20** | 4,267 | 24,038 | 13,440 | 7,656 |
| **N25** | 3,773 | 33,206 | 11,745 | 10,602 |
| **N30** | 3,365 | 43,523 | 10,311 | 13,964 |
| **N35** | 3,030 | 55,028 | 9,103 | 17,781 |
| **N40** | 2,728 | 67,803 | 8,082 | 22,094 |
| **N45** | 2,461 | 81,974 | 7,145 | 26,952 |
| **N50** | **2,224** | 97,670 | **6,289** | 32,467 |
| **N55** | 2,002 | 115,070 | 5,494 | 38,753 |
| **N60** | 1,799 | 134,426 | 4,748 | 45,985 |
| **N65** | 1,608 | 156,014 | 4,038 | 54,416 |
| **N70** | 1,425 | 180,261 | 3,361 | 64,430 |
| **N75** | 1,248 | 207,782 | 2,703 | 76,665 |
| **N80** | 1,076 | 239,415 | 2,060 | 92,292 |
| **N85** | 904 | 276,648 | 1,462 | 113,532 |
| **N90** | 703 | 322,528 | 982 | 144,672 |
| **N95** | 479 | 384,742 | 594 | 193,132 |
| **N100** | 24 | 498,189 | 200 | 287,478 |
| **Total** | 734,172,073 | 498,189 | 739,042,261 | 287,478 |

**Table S6** List of species used in this study for phylogenetic analyses and for genomic content analysis

| **Species** |  | **acc num mitogenome** | **SRA/BioProject** |
| --- | --- | --- | --- |
| *Cacopsylla_melanoneura* | Psyllidae | PX243765 | PRJNA1312285 |
| *Cacopsylla_picta* | Psyllidae | PX243766 | PRJNA1312285 |
| *Cacopsylla_pyri* | Psyllidae | PV137636 | PRJNA853726 |
| *Cacopsylla_pyricola* | Psyllidae | PV137637 | PRJNA853282 |
| *Cacopsylla_mali* | Psyllidae | PV137633 | PRJNA1211559 |
| *Cacopsylla_pruni* | Psyllidae | PV137635 | PRJNA1211557 |
| *Cacopsylla_pyrisuga* | Psyllidae | PV137638 | PRJNA1211560 |
| *Cacopsylla_burckhardti* | Psyllidae | NC_069642.1 | SRX15496259 |
| *Cacopsylla_jukyungi* | Psyllidae | NC_069847.1 | SRX16308894 |
| *Cacopsylla_coccinea* | Psyllidae | NC_027087.1 | - |
| *Cacopsylla_fuscicella* | Psyllidae | NC_080374.1 | - |
| *Cacopsylla_citrisuga* | Psyllidae | NC_053749.1 | - |
| *Heteropsylla cubana* | Psyllidae | PV137639 | - |
| *Diaphorina citri* | Psyllidae | MF426268.1 | - |
| *Euphyllura phillyreae* | Liviidae | NC_038134.1 | - |
| *Livia junci* | Liviidae | NC_038137.1 | - |
| *Paurocephala sauteri* | Liviidae | NC_053635.1 | - |
| *Melanastera paucipunctata* | Liviidae | NC_038109.1 | - |
| *Pachypsylla venusta* | Carsidaridae | AY278317.1 | - |
| *Lanthanaphalara mira* | Aphalaridae | NC_038111.1 | - |
| *Aphis gossypii* | Aphididae | NC_024581.1 | - |
| *Myzus persicae* | Aphididae | NC_029727.1 | - |
| *Bemisia tabaci* | Aleyrodidae | NC_006279.1 | - |
| *Aleurodicus dugesii* | Aleyrodidae | NC_005939.1 | - |

**
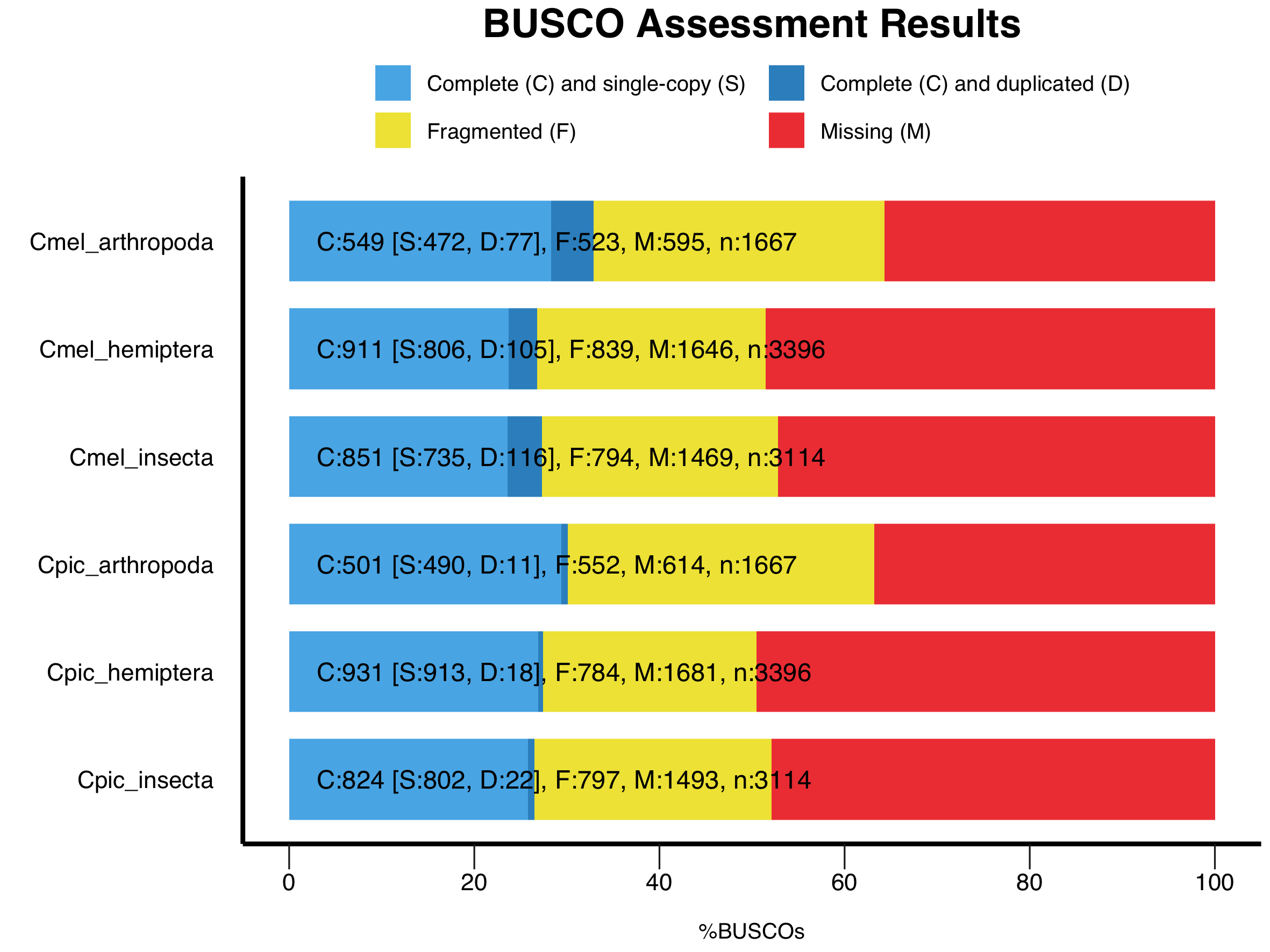
**

Figure S1. BUSCO assessment results. Number of complete, fragmented and missing BUSCO orthologous genes in the *C. melanoneura* (Cmel) and *C. picta* (Cpic) assembled genomes. The analyis is based on the odb12 databases for Arthropoda, Insecta and Hemiptera.


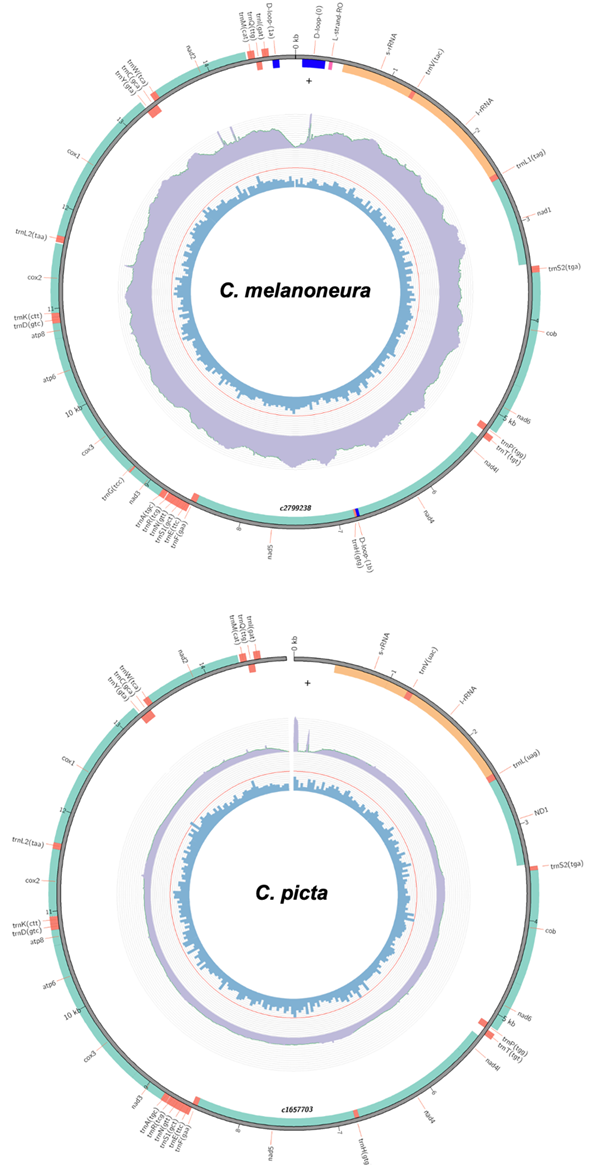


Figure S2. Circular representations of complete *Cacopsylla melanoneura* and *C. picta* mitochondrial genomes. The position and orientation of the 13 PCG genes (green), 22 tRNA genes (red), 2 rRNA genes (orange), control region (blue) and origin of L-strand replication (pink) are shown. The inner circle displays GC bias and the outer circle displays coverage depth of the Illumina reads used mitochondrial genome assembly.


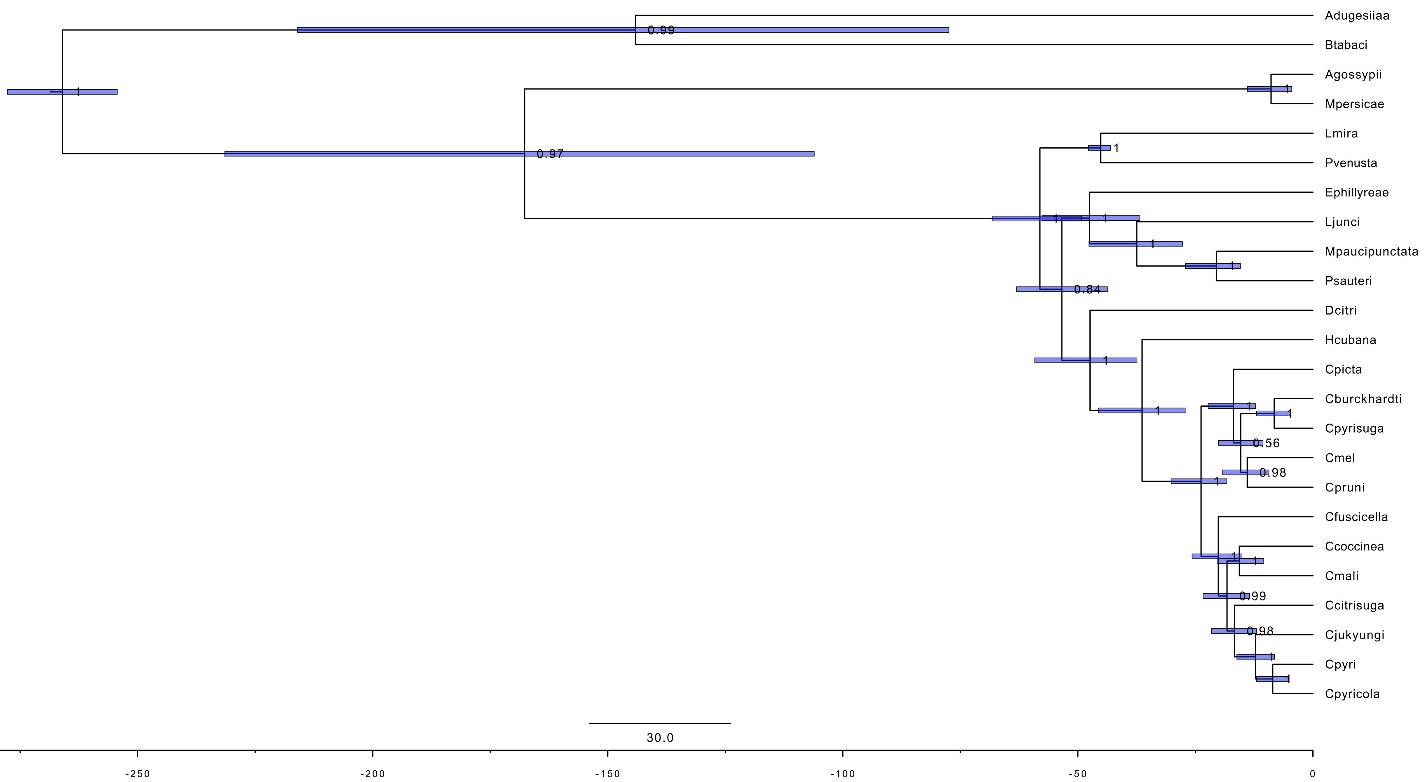


Figure S3. Bayesian tree with nucleotides 123


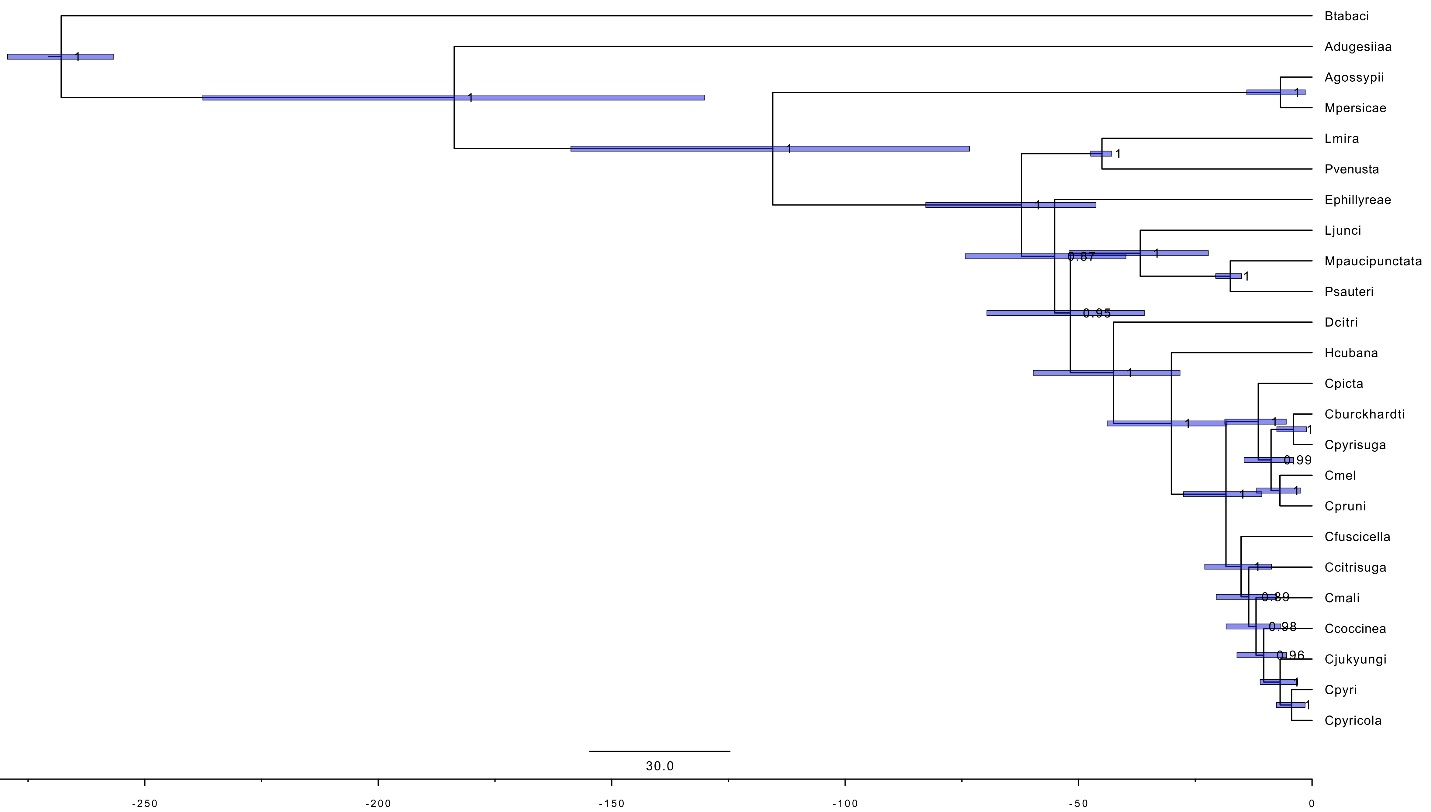


Figure S4. Bayesian tree with amino acids


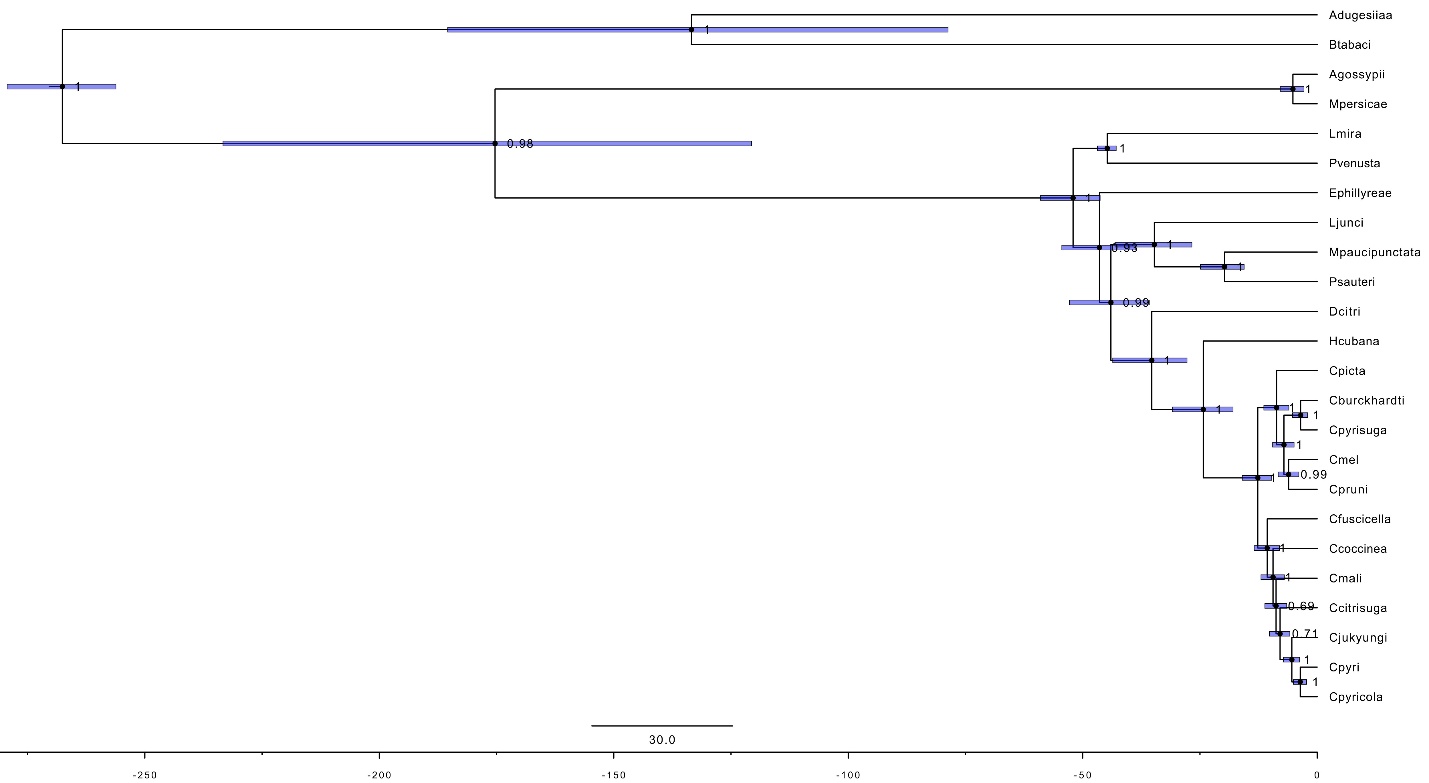


Figure S5. Bayesian tree without 3d codon positions


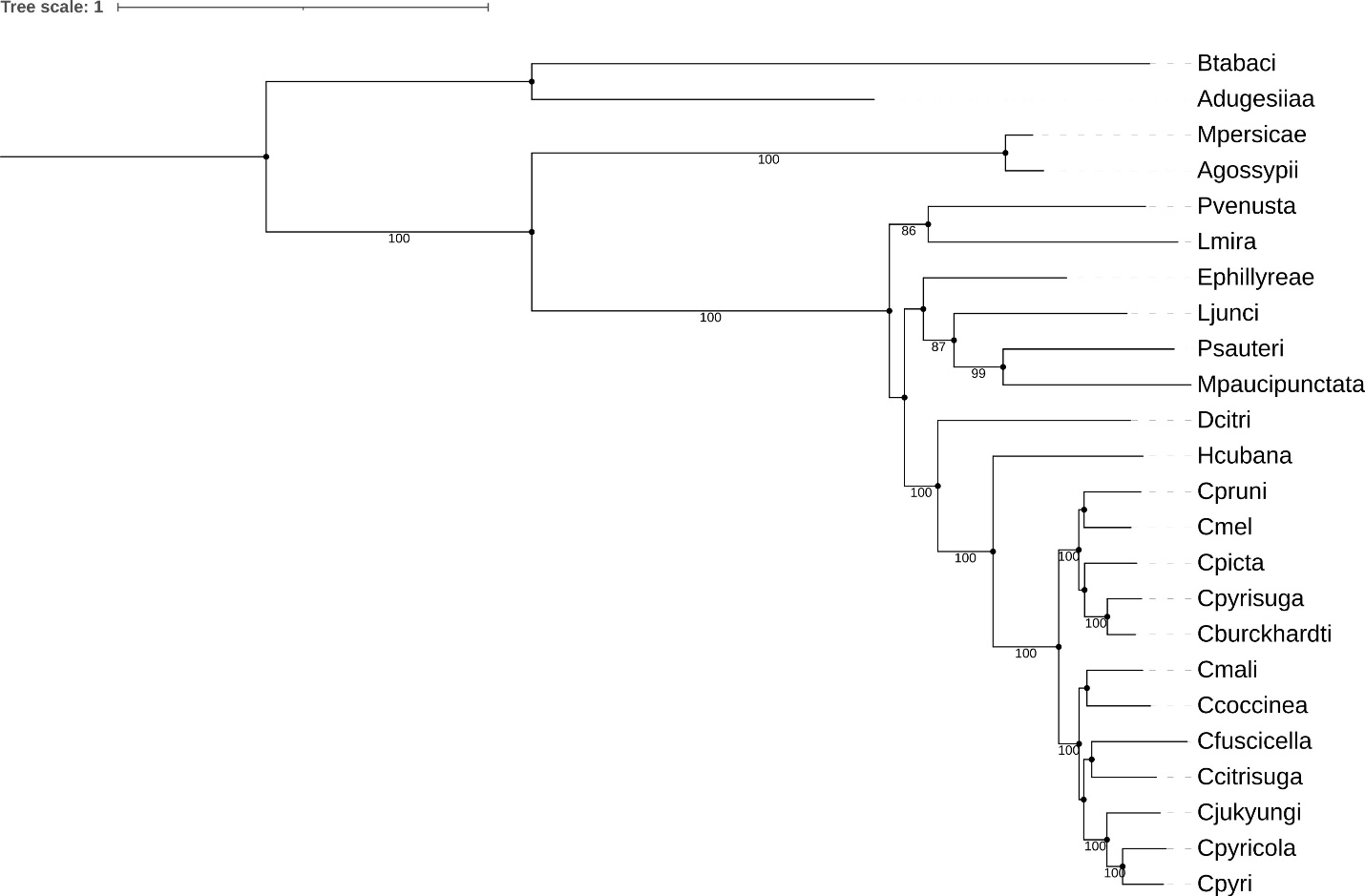


Figure S6. Maximum likelihoods with nucleotides 123


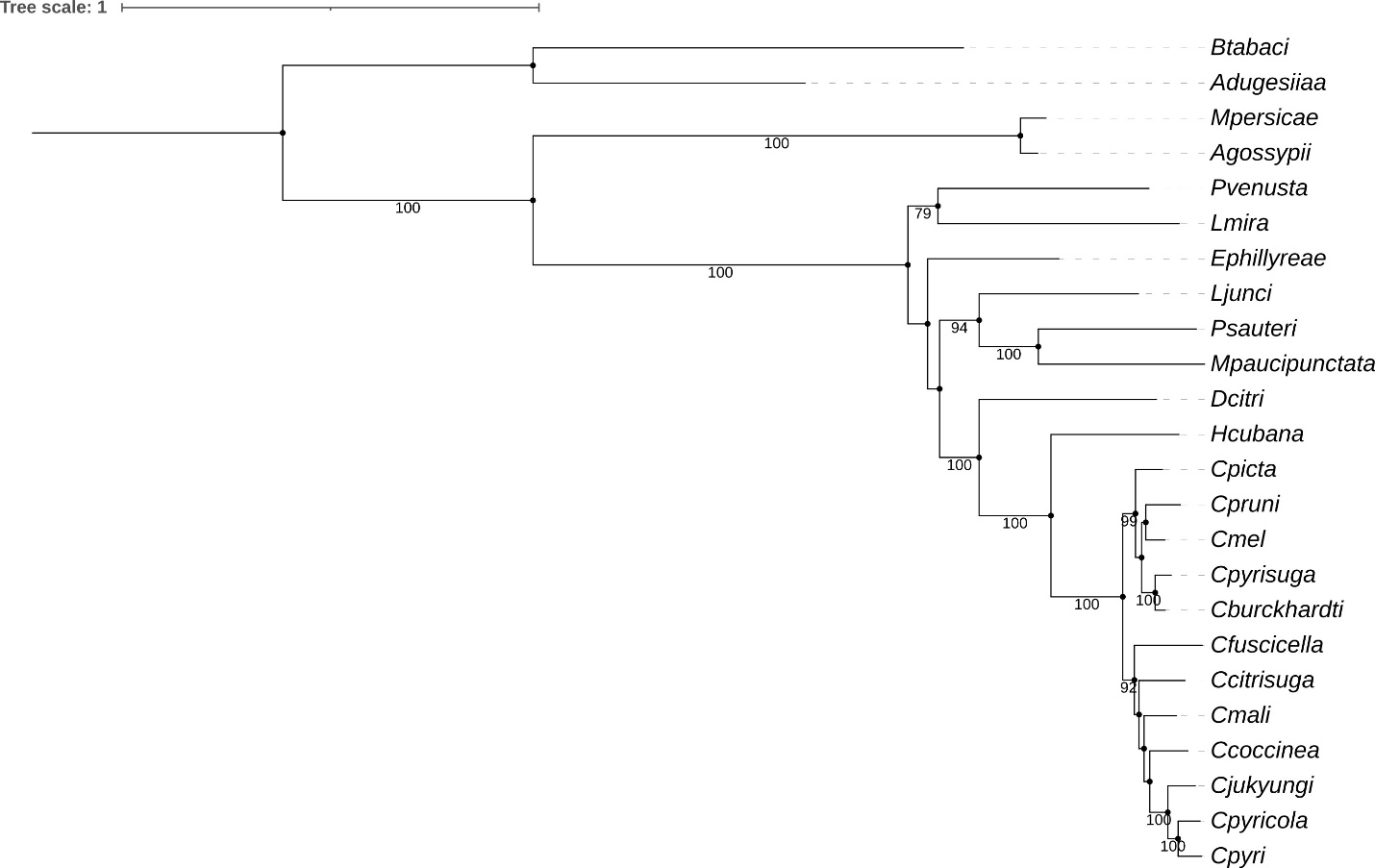


Figure S7. Maximum likelihoods tree with amino acids


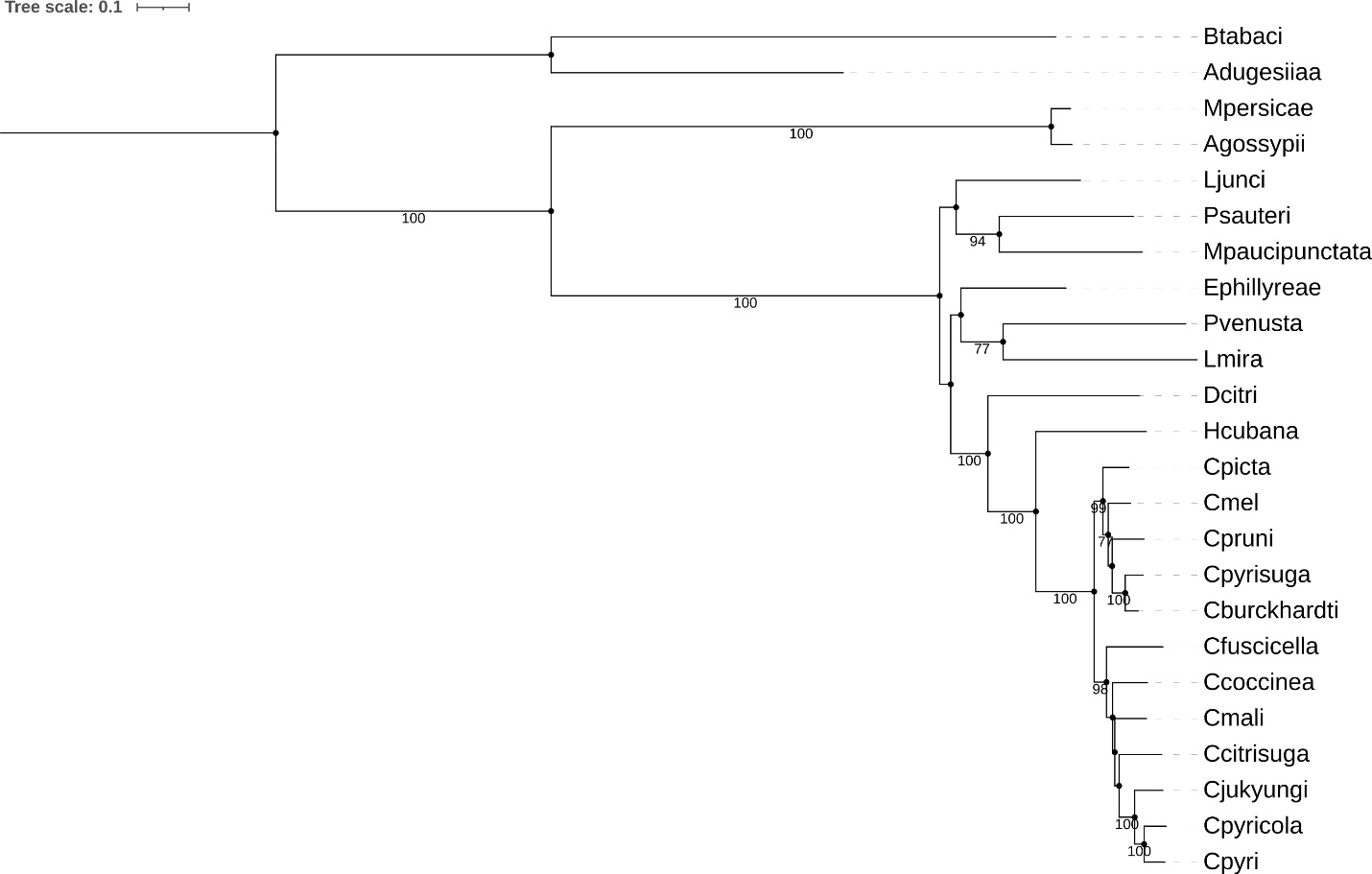


Figure S8. Maximum likelihoods tree without 3d codon positions.


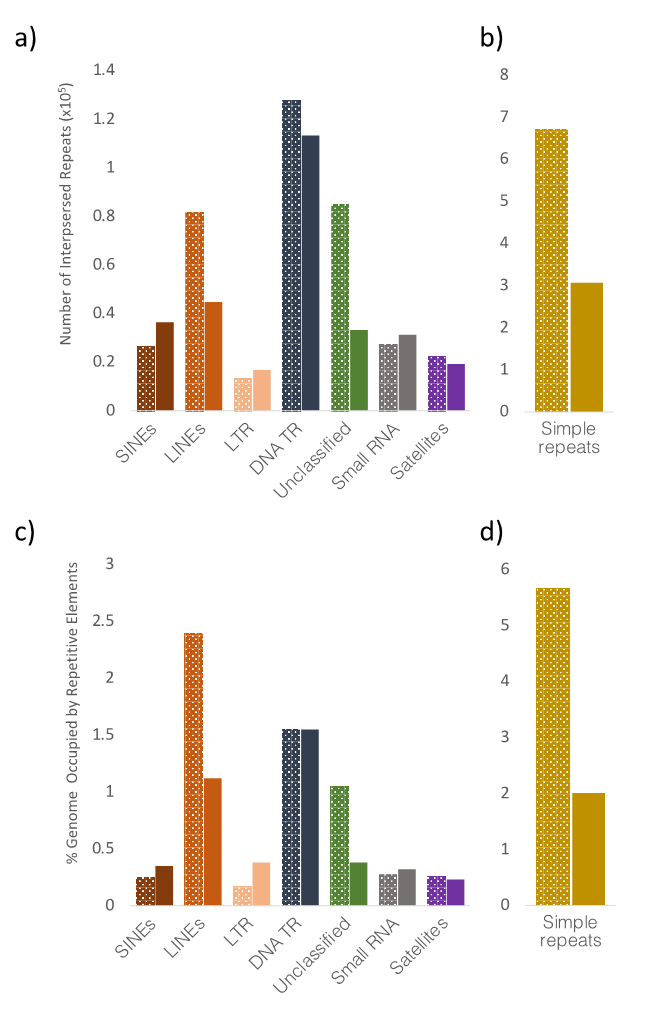


Figure S9. Bar plots showing the count frequency (a,b) and percentage of genome occupied by various classes of genomically interspersed repeats (c,d), including retroelements (SINE, LINE, LTR; a,c), DNA transposons, unclassified repetitive elements, sRNAs and satellites, and simple repeats, in *Cacopsylla picta* (dotted bars) and in *C. melanoneura* (solid bars (b,d).


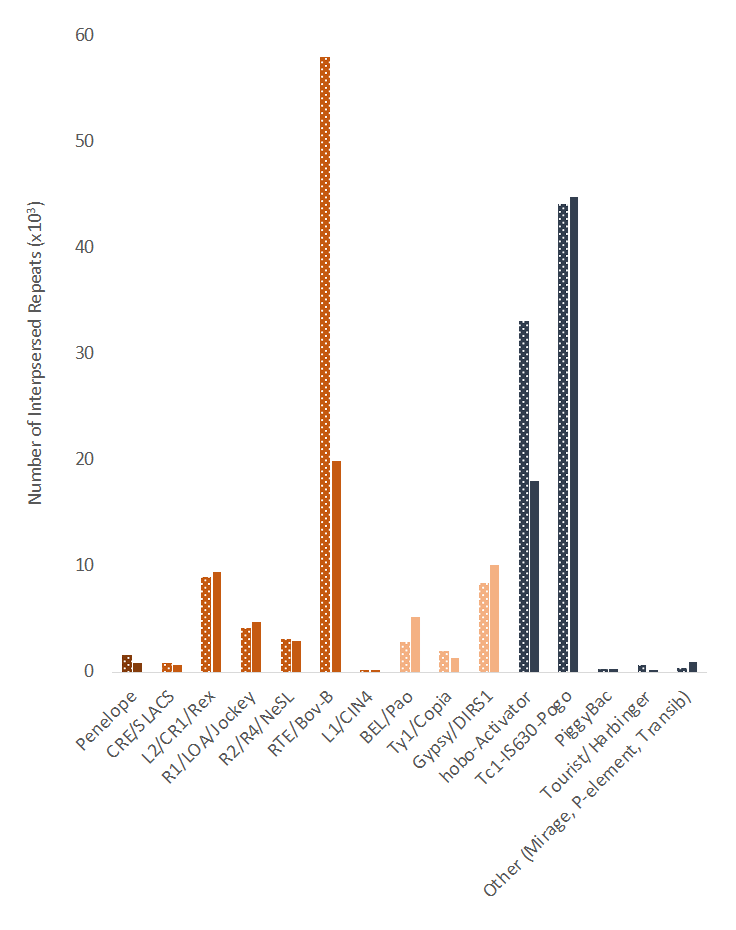


Figure S10. Bar plots showing count frequencies of specific genomically interspersed repetitive elements in *Cacopsylla picta* (dotted bars) and *C. melanoneura* (solid bars). Colours correspond to repetitive element classes. Retroelements of class SINE are in brown, of class LINE in ochre orange, and class LTR in salmon. DNA transposons are in slate grey.
